# Occupational sleep restriction: a missing piece in residual EDS of PAP-adherent OSA?

**DOI:** 10.1007/s44470-026-00153-0

**Published:** 2026-08-24

**Authors:** Christoph G.U. Riese, Ulrich Koehler

**Affiliations:** Schlafmedizinisches Zentrum, Philipps-Universität Marburg, Marburg, Germany

We read with interest the MAGNETO study by Junco et al., demonstrating that residual excessive daytime sleepiness (EDS) in PAP-adherent obstructive sleep apnea (OSA) is associated with cognitive deficits [1]. The authors adjusted for sex, diagnostic latency, and sleep duration, yet these covariates did not fully explain the cognitive differences. What drives persistent EDS in patients with adequately controlled apnea?

In our cohort of 543 treatment-naïve OSA patients, employed participants had significantly higher Epworth Sleepiness Scale scores than non-employed participants (11.4 ± 4.8 vs. 9.7 ± 4.7; *p* < 0.001), despite identical apnea severity (AHI 37.8 vs. 39.1; *p* = 0.27). Weekly rest time was 68 min shorter in employed patients (443 vs. 511 min; *p* < 0.001), yielding a cumulative sleep debt of 82 min versus 13 min. The European Sleep Apnea Database similarly reported approximately 28% residual EDS despite adequate CPAP, attributed to cultural and lifestyle factors [2].

The pathway from employment to EDS extends beyond time deficit. A meta-analysis of 201 studies confirmed that calling drives longer work hours and poorer psychological detachment, reducing sleep quality independently of duration [3, 4]. This mechanism, documented in high-commitment populations, remains unexamined in OSA. Reduced psychological detachment has been linked to impaired cognitive performance [5], suggesting a pathway from occupational sleep restriction to the cognitive deficits observed by Junco et al.

Junco et al. identified residual EDS as a cognitive risk phenotype but did not account for occupational factors. Our data suggest that work-related sleep debt and impaired psychological detachment may contribute to this phenotype. If confirmed, cognitive deficits in residual EDS may partly reflect occupational sleep restriction rather than inadequately treated apnea alone. Before escalating to wake-promoting agents, clinicians might first assess occupational sleep hygiene.

We recommend that prospective studies on residual EDS in PAP-adherent OSA include validated measures of work hours, shift schedules, sleep debt, psychological detachment, and prosocial work orientation. Such assessments would clarify the contribution of occupational factors to heterogeneity in EDS phenotypes and identify a modifiable target that may reduce both sleepiness and its cognitive consequences.

## Data Availability

Data available from the corresponding author upon reasonable request.
